# PSYCHOTIC PATIENT AND AFFECTATION OF THE SEXUAL SPHERE. ABOUT A CLINICAL CASE

**DOI:** 10.1192/j.eurpsy.2023.2314

**Published:** 2023-07-19

**Authors:** S. Susana Perez, I. Martin-Herrero

**Affiliations:** Psychiatry, Los Arcos del Mar Menor Universitary Hospital, Murcia, Spain

## Abstract

**Introduction:**

Clinical case description.

A 44-year-old male, with paranoid schizophrenia, polydrug addict and IVDU since he was 14 years old. Live alone. He is brought by the Local Police for heteroaggressiveness when he was arrested for trespassing and resisting authority. He has presented 14 hospital admissions and therapeutic communities for detoxification since 2000.

**Objectives:**

1. To analyze the causes of abandonment of treatment

**Methods:**

1. Complete medical history
2. Blood test with hormone profile
3. PRAEQ Questionnaire

Absence of child psychopathology until the age of 6, at which time his parents separate and he becomes introverted, with solitary activities and distractions. On examination, the patient is restless. Psychotic contact. Delusional speech centered on ill-structured ideas of harm, persecution and grandiose, being difficult to explore due to lack of collaboration on the part of the patient, who only accepts treatment with aripiprazole 5 mg due to sexual dysfunction with the rest of the treatments.

**Results:**

During his admission, he has evolved from a very unstructured delusional theme and social isolation, with lax and tangential speech and refusal to take any treatment except aripiprazole, towards a cooperative, trusting attitude, with attenuation of his delusional ideation, with appropriate, organized and releasable. Treatment with IM aripiprazole is agreed upon for discharge.

Clinical judgement: Paranoid schizophrenia (F20.0)

**Conclusions:**

With this case, we intend to remember that antipsychotics affect different spheres of the patient’s life that can hinder adherence to treatment, and that we often do not take into account. In this specific case, Abilify Maintena is useful because it does not cause sexual dysfunction, which facilitates treatment adherence and greater patient involvement, which gives us greater opportunities for social integration.

**Disclosure of Interest:**

None Declared

